# Human granulocytic anaplasmosis combined with rhabdomyolysis: a case report

**DOI:** 10.1186/s12879-021-06869-z

**Published:** 2021-11-25

**Authors:** Jeong Min Cho, Jeonghyun Chang, Dong-Min Kim, Yee Gyung Kwak, Chong Rae Cho, Je Eun Song

**Affiliations:** 1 Division of Infectious Disease, Department of Internal Medicine, Inje University Ilsan Paik Hospital, 170, Juhwa-ro, Ilsanseo-gu, Goyang-si, Gyeonggi-do Republic of Korea; 2 Department of Laboratory Medicine, Inje University Ilsan Paik Hospital, Goyang, Republic of Korea; 3 Department of Internal Medicine, Chosun University Hospital, Gwangju, Republic of Korea

**Keywords:** Human granulocytic anaplasmosis, Rhabdomyolysis, Tick-borne diseases, *Anaplasma phagocytophilum*, Case report

## Abstract

**Background:**

Human granulocytic anaplasmosis (HGA) is a systemic inflammatory response caused by the rickettsial bacterium *Anaplasma phagocytophilum*. Rhabdomyolysis and acute kidney injury (AKI) are rare complications of HGA. Here, we report a case of HGA concurrent with rhabdomyolysis and AKI in an elderly patient.

**Case presentation:**

An 84-year old woman with a medical history of hypertension was hospitalised after two days of fever, dizziness, whole body pain, and general weakness. Laboratory investigations showed severe thrombocytopenia, leukopenia, impaired renal function, and elevated cardiac enzyme and myoglobin levels. On the day after admission, peripheral blood smear revealed morula inclusions in neutrophils, a suggestive finding of HGA. Real-time polymerase chain reaction (PCR) results indicated the presence of *A. phagocytophilum*. Antibiotics were de-escalated to doxycycline monotherapy. After 10 days of antibiotic treatment, laboratory tests showed complete recovery from HGA complicated with rhabdomyolysis and AKI.

**Conclusions:**

HGA can lead to serious complications in patients with associated risk factors. Therefore, in patients with HGA accompanied by rhabdomyolysis, management with antibiotics and hydration should be initiated immediately, and not delayed until diagnostic confirmation.

## Background

Human granulocytic anaplasmosis (HGA) is an ixodid tick-borne zoonosis caused by the rickettsial bacterium *Anaplasma phagocytophilum* [1]. Since its first report in the United States in 1994, the incidence of HGA has steadily increased. The first case of HGA was reported in 2013 in the Republic of Korea (ROK, South Korea) [2]. Acute febrile illness develops after granulocytes are infected by ixodid ticks. Clinical manifestations include high fever, chills, myalgia, headache, nausea, arthralgia, and dry cough, with an incubation period of approximately 7–10 days after being bitten by an infected tick [3]. Typical laboratory findings include leukopenia, thrombocytopenia, and elevated transaminase levels [4]. More severe outcomes, including septic shock, multi-organ failure and death can occur in immunocompromised patients [3]. Confirmatory diagnostic tests include peripheral blood smear (PBS) to detect morula in the cytoplasm of infected circulating granulocytes, serological assays such as indirect immunofluorescence antibody, and real-time polymerase chain reaction (PCR) [5]. The difficulty with HGA is that clinical symptoms are nonspecific, often mimicking a viral illness [1]. In particular, when accompanied by rhabdomyolysis, early suspicion and diagnosis are important, as sufficient intravenous hydration is important. Here, we report the first case of HGA accompanied by rhabdomyolysis in an elderly patient diagnosed with PBS and PCR in Korea.

## Case presentation

An 84-year-old woman with a history of hypertension presented in our hospital after two days of fever and dizziness. She lived in Dangjin-gun, Chungcheongnam-do Province, ROK; her house was located at the base of a mountain, and she often worked in her yard. She was transferred to our hospital because of fever, thrombocytopenia, elevated cardiac enzyme levels and creatinine levels. Upon arrival, her body temperature was 36.0 °C, blood pressure was 92/53 mmHg, and pulse rate was 70 beats per minute. No eschar or skin rash was found on physical examination. Initial laboratory investigations showed a normal leukocyte count of 4.3 × 10^3^/µL and decreased platelet count of 34 × 10^3^/µL. Liver enzyme alanine aminotransferase and aspartate aminotransferase levels were elevated to 188 and 55 IU/L, respectively. The levels of cardiac enzymes, including creatinine kinase (CK), CK-MB, and troponin I, were increased to 3107, 22.8 ng/mL, and 122.2 pg/mL, respectively. The serum myoglobin level, which indicates the possibility of rhabdomyolysis, was elevated to over 1200 ng/mL. Serum blood urea nitrogen (BUN) and creatinine levels were elevated to 58.0 and 2.41 mg/dL, respectively, and the estimated glomerular filtration rate decreased to 19.1. C-reactive protein (CRP) levels were elevated to 17.4 mg/dL.

**Fig. 1:**
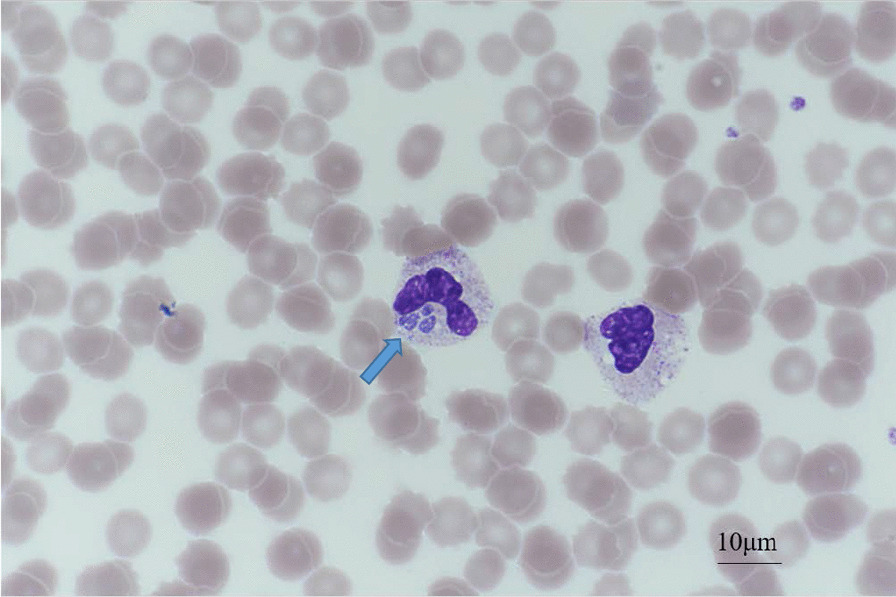
*Anaplasma phagocytophilum* morula inclusion (arrow) in peripheral white blood cells. (× 1000) Photographs were taken with Nikon Y-TV55 camera and Nikon NIS Elements imaging software

One day after hospitalisation, her body temperature increased to 39.4 °C, which was accompanied by severe chill sensations, and the pulse rate increased to 120 beats per minute. Electrocardiography (ECG) was performed and new-onset atrial fibrillation was observed. After administering amiodarone, the patient’s ECG returned to the sinus rhythm. The levels of CK, CK-MB, and troponin I remained elevated at 2882, 19.7 ng/mL, and 87.1 pg/mL, respectively. Transthoracic echocardiography was performed to rule out myocardial infarction and the results were normal. A thick PBS was examined for the presence of *A. phagocytophilum* in the neutrophils (Nikon Y-TV55). On PBS, morula inclusions in neutrophils suggestive of HGA (Fig. 1) were shown.

We administered 100 mg of doxycycline orally twice a day and 2 g of ceftriaxone intravenously once a day. On the third day of hospitalisation, the patient presented with an improved condition and appetite. Her serum levels of cardiac enzymes, BUN, creatinine, and liver enzymes had improved significantly. Antibiotics were de-escalated to doxycycline monotherapy. However, white blood cell (WBC) and platelet counts continued to decrease to 1.78 × 10^3^/µL and 17 × 10^3^/µL on the third and fourth day of admission, respectively. After four days of hospitalisation, leukopenia and thrombocytopenia were ameliorated.

**Fig. 2:**
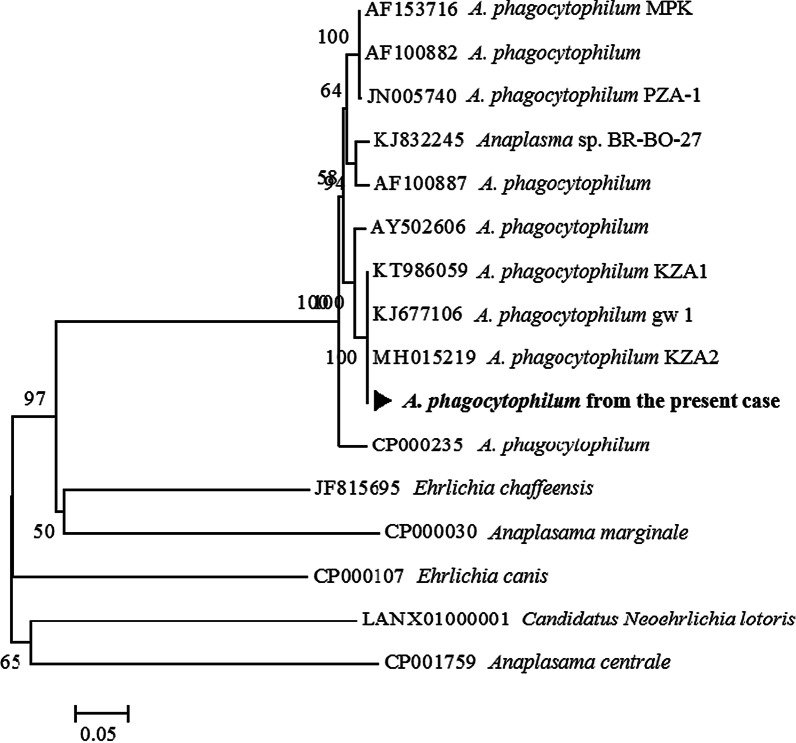
Phylogenetic relationships of *Anaplasma phagocytophilum* from the present case (NCBI accession no. OL265754). The tree was analysed after *AnkA* nested PCR

Blood samples were tested by performing nested PCR with *Anaplasma specific* primers targeting the ankyrin-repeat protein AnkA gene (*AnkA*). A portion of the 16S ribosomal RNA gene (16S rRNA) was amplified by PCR. PCR amplicons were purified and directly sequenced using PCR primers. Basic Local Alignment Search Tool (BLAST) analysis of the sequenced products confirmed *A. phagocytophilum* infection. The 564 bp *AnkA* gene sequence (NCBI accession no. OL265754) was 99% identical to that of *A. phagocytophilum* KZA2 (accession no. MH015219) (Fig. 2). Other blood tests were negative for hantavirus, severe fever thrombocytopenia syndrome virus, *Orientia tsutsugamushi*, and leptospirosis.

On the ninth day of hospitalisation, laboratory tests showed that WBC and platelet counts had normalised to 10.34 × 10^3^/µL and 169 × 10^3^/µL, respectively. Serum CRP and CK levels were decreased to 1.2 mg/dL and 42 IU/L. The patient was discharged after a further three days of doxycycline for a total of 10 days of antibiotic treatment. A week later, she was evaluated in the outpatient unit and found to have recovered completely from renal insufficiency, rhabdomyolysis, and cytopenia.

## Discussion and conclusions

HGA is a zoonotic tick-borne disease caused by the intracellular bacterium, *A. phagocytophilum*. The incidence of HGA cases has been increasing steadily in the United States since they were first reported in 2000 [6]. The first case of HGA in ROK was reported in 2014 [2]. However, a retrospective study analysing 16S rRNA genes of *A. phagocytophilum* in patients with unknown causes of fever and cytopenia from 2003 through 2012 revealed that 5 of 70 patients (7.1%) were consistent with those of HGA since 2006 [7]. The differential diagnosis of HGA might not have been accomplished properly due to limited knowledge regarding clinical manifestations and laboratory tests available for HGA diagnosis.

Human granulocytes become infected when transmitted by ixodid ticks, including *Ixodes scapularis, Ixodes pacificus* and *Ixodes ricinus.* Intracellular inclusion called morulae were found in the microscopic examination of Giemsa-stained smears. Morulae which appear as stippled blue inclusions of bacteria in monocytes or neutrophils and bands, form the most rapid diagnostic tool that can be used after disease onset [8]. Morulae tend to be detected more frequently within the first week of the disease; therefore, the absence of morulae does not exclude the diagnosis of HGA [9].

Although HGA is usually a self-limiting disease, it can progress to serious complications such as acute respiratory distress syndrome, disseminated intravascular coagulopathy, rhabdomyolysis, and acute kidney injury (AKI) in certain patients. Patient age, immunocompromised status, comorbidities, and a delay in diagnosis and treatment are known risk factors for poor prognosis of HGA [9, 10]. In our case, the patient had at least two risk factors: hypertension and old age, which may have contributed to the more severe course of rhabdomyolysis.

Rhabdomyolysis and AKI are rare complications of HGA. To the best of our knowledge, this is the first case reported in the ROK, while only four cases have been previously reported in the United States [11–14]. In these previous reports, patients were over 65 years of age in three cases, and statins were concomitantly used in one case. In all cases, rhabdomyolysis and AKI secondary to HGA spontaneously improved with conservative treatment. The CK levels ranged from 3000 to 150,000 U/L.

The aetiology and mechanism of rhabdomyolysis caused by HGA complications remain unknown. Trauma, drugs, and infections are the most commonly reported causes of rhabdomyolysis in adults [15]. The mechanisms underlying rhabdomyolysis caused by infections include hypoxia, down- or upregulated enzymatic responses, and endotoxic effects [16]. Neutrophil adhesion to endothelial cells is an important process in tissue inflammation. However, *A. phagocytophilum*-infected cells show reduced adherence to endothelial cell lines [17]. Instead, increased release of cytokines and activation of macrophages are considered to be associated with HGA-induced rhabdomyolysis [18].

Although HGA is not widely known in the ROK, anaplasmosis should always be considered in the differential diagnosis of febrile illness and other infectious diseases. In addition to other clinical manifestations, rhabdomyolysis should be considered as supporting data for diagnosing HGA. In patients whose clinical features and laboratory tests are suggestive of HGA with rhabdomyolysis, antibiotic treatment and hydration should be initiated immediately, rather than being delayed until diagnostic confirmation. In our case, immediate clinical management, which is correlated with a better prognosis, enabled the patient to recover completely.

In conclusion, we report a rare case of HGA-associated rhabdomyolysis that was completely ameliorated by the use of antibiotics. Considering the increasing prevalence of tick-borne disease, suspecting rickettsiosis including anaplasmosis and conducting immediate diagnostic tests and management would lead to a more positive prognosis for patients.

## Data Availability

All data generated or analysed during this study are included in this published article.

## Abbreviations

BLAST: Basic local alignment search tool
BUN: Blood urea nitrogen
CK: Creatinine kinase
CRP: C-reactive protein
ECG: Electrocardiography
HGA: Human granulocytic anaplasmosis
PBS: Peripheral blood smear
PCR: Polymerase chain reaction
ROK: Republic of Korea
WBC: White blood cell
